# Associating brain imaging phenotypes and genetic risk factors *via* a hypergraph based netNMF method

**DOI:** 10.3389/fnagi.2023.1052783

**Published:** 2023-03-02

**Authors:** Junli Zhuang, Jinping Tian, Xiaoxing Xiong, Taihan Li, Zhengwei Chen, Rong Chen, Jun Chen, Xiang Li

**Affiliations:** ^1^Department of Vascular Surgery, Renmin Hospital of Wuhan University, Wuhan, China; ^2^Faculty of Medicine, Jianghan University, Wuhan, China; ^3^Central Laboratory, Renmin Hospital of Wuhan University, Wuhan, China; ^4^Department of Clinical Laboratory, The First Affiliated Hospital of Shenzhen University, Shenzhen Second People's Hospital, Shenzhen, China; ^5^Department of Radiology, Hubei Provincial Hospital of Traditional Chinese Medicine, Wuhan, China; ^6^School of Health, Wuhan University, Wuhan, China

**Keywords:** non-negative matrix factorization, Alzheimer’s disease, mild cognitive impairment, hypergraph learning, biomarkers

## Abstract

**Abstract:**

Alzheimer’s disease (AD) is a severe neurodegenerative disease for which there is currently no effective treatment. Mild cognitive impairment (MCI) is an early disease that may progress to AD. The effective diagnosis of AD and MCI in the early stage has important clinical significance.

**Methods:**

To this end, this paper proposed a hypergraph-based netNMF (HG-netNMF) algorithm for integrating structural magnetic resonance imaging (sMRI) of AD and MCI with corresponding gene expression profiles.

**Results:**

Hypergraph regularization assumes that regions of interest (ROIs) and genes were located on a non-linear low-dimensional manifold and can capture the inherent prevalence of two modalities of data and mined high-order correlation features of the two data. Further, this paper used the HG-netNMF algorithm to construct a brain structure connection network and a protein interaction network (PPI) with potential role relationships, mine the risk (ROI) and key genes of both, and conduct a series of bioinformatics analyses.

**Conclusion:**

Finally, this paper used the risk ROI and key genes of the AD and MCI groups to construct diagnostic models. The AUC of the AD group and MCI group were 0.8 and 0.797, respectively.

## 1. Introduction

Alzheimer’s disease (AD) is a neurodegenerative disease with insidious onset and progressive development. The most common early symptom is difficulty remembering recent events (Scheltens et al., 2021). As the disease progresses, patients gradually lose their ability to care for themselves and eventually die from complications such as infection (Scheltens et al., 2021). The exact cause of AD is still unknown, but it has a long-standing preclinical feature: mild cognitive impairment (MCI). The typical symptoms of MCI patients are memory loss and there may be damage to one or more cognitive areas (Carlson and Prusiner, 2021). Still, it is not enough to affect the patient’s daily life, and the diagnostic criteria for AD have not yet been met. Previous studies show that structural and functional abnormalities of the brain and phenotypic or molecular abnormalities associated with AD are associated (Simrén et al., 2021). However, the source or cause of these abnormalities is unclear.

In recent years, imaging genetic analysis related to brain diseases has attracted much attention. Imaging genetics explores the effect of genetic variation on brain structure, metabolism, and function through association analysis of radionics data (such as sMRI, PET, fMRI; Jiang et al., 2018) and genetic data (such as DNA methylation, SNP, gene expression; Du et al., 2020).

The association mechanism of imaging genetics is gradually revealed through robust association analysis algorithms. By improving sparse canonical correlation analysis (SCCA) with various strategies, Du et al. (2020) proposed various innovative algorithms based on SCCA to analyze brain imaging genetics data. For example, they proposed a dirty multi-task sparse canonical correlation analysis (dirty MT-SCCA) to study imaging genetic problems involving multimodal brain imaging. This method used multi-task learning and parameter decomposition to simultaneously identify pattern-consistent and pattern-specific brain regions and SNP loci. Furthermore, they proposed a parametric decomposition-based sparse multi-view canonical correlation analysis (PDSMCCA) method to identify modality-sharing and specific information from multimodal data to gain insight into the complex pathology of brain diseases (Zhang et al., 2022). However, the above two algorithms only supported the analysis of multimodal brain imaging and single-modal genetic data. The sparse multi-view sparse canonical correlation analysis (SMCCA) algorithm can simultaneously perform correlation analysis on data from multiple modalities. However, the direct fusion of multiple SCCA objectives can cause gradient domination problems, resulting in SMCCA being a suboptimal model. Therefore, they proposed two adaptive multi-view canonical correlation analysis algorithms to solve the problem of gradient domination, integrating the underlying relationships between protein expression, SNPs, and neuroimaging (Du et al., 2021).

On the other hand, multi-objective optimization algorithms are also emerging in the field of imaging genetics. Bi et al. (2022) integrated the fMRI and SNP data of Parkinson’s patients through multiple optimization methods for multimodal analysis. They used a correlation analysis method to construct fused features from the sequence information and SNPs of regions of interest (ROIs). Then, a weighted evolution strategy was introduced into ensemble learning, and a new weighted evolutionary random forest (WERF) model was constructed to eliminate inefficient features. In addition, they also proposed a clustered evolutionary random forest (CERF) method to detect discriminative genes and brain regions and found some interesting associations between brain regions and genes in Parkinson’s patients (Bi et al., 2021).

However, the above algorithms cannot interpret the results from the perspective of network regulation. Therefore, this paper used a hypergraph-based netNMF (HG-netNMF) algorithm to explore the relationship between genes and sMRI from both gene network and brain network construction. The algorithm exploited hypergraph regularization to mine higher-order features of both modal data. Further, the key genes and ROIs were explored from the critical network modules. Bioinformatics analysis and diagnostic model construction were performed to provide new insights into the imaging genetic association mechanism of AD and MCI.

## 2. Methods

### 2.1. Nonnegative matrix factorization

Nonnegative matrix factorization (NMF) algorithm is a classic dimensionality reduction algorithm that were widely used in image processing, audio processing, biological data analysis, and other fields (Kim and Tidor, 2003; Zhang et al., 2012; Yang et al., 2021). It decomposes the matrix $X\in {\mathbb{R}}^{n\times p}$ to obtain the basis matrix $W\in {\mathbb{R}}^{n\times k}$ and the coefficient matrix $H\in {\mathbb{R}}^{k\times p}$, and its objective function is as follows:


(1) $$\min_{W,H}∥X-WH{∥}_{F}^{2}$$

### 2.2. Network non-negative matrix factorization

The traditional NMF algorithm can only decompose single-modal data and cannot analyze the network control module composed of multi-modal data. When X is a symmetric similarity matrix, Chen and Zhang (2018) proposed the netNMF algorithm to decompose multiple matrices simultaneously. It is worth noting that the algorithm can be used to analyze the network modules of various data. For example, their respective correlation networks and mutual correlation networks can be calculated for analysis for the two expression matrices $X_{1}\in {\mathbb{R}}^{n\times p}$, $X_{2}\in {\mathbb{R}}^{n\times q}$. The objective function of the netNMF algorithm is as follows:


(2) $$\min_{G_{1},G_{2},S_{11},S_{22}}{\left\|R_{11}-G_{1}S_{11}G_{1}^{T}\right\|}_{F}^{2}+{\left\|R_{12}-G_{1}G_{2}^{T}\right\|}_{F}^{2}+{\left\|R_{22}-G_{2}S_{22}G_{2}^{T}\right\|}_{F}^{2}\text{s.t.}G_{1},G_{2},S_{11},S_{22}\geq 0$$

Among them, $R_{11}\in {\mathbb{R}}^{p\times p}$, $R_{22}\in {\mathbb{R}}^{q\times q}$ are the respective association networks of $X_{1}$ and $X_{2}$. $R_{12}\in {\mathbb{R}}^{p\times q}$ is the cross-correlation network of $X_{1}$ and $X_{2}$. $G_{11}\in {\mathbb{R}}^{p\times k}$, $G_{22}\in {\mathbb{R}}^{q\times k}$, $S_{11}\in {\mathbb{R}}^{k\times k}$, $S_{22}\in {\mathbb{R}}^{k\times k}$ are non-negative factor matrices. The algorithm can be analyzed from the network module level constructed by the data, and $R_{12}-G_{1}G_{2}^{T}{}_{F}^{2}$ is used to identify the one-to-one relationship between the two types of modules.

In fact, $R_{11}\in {\mathbb{R}}^{p\times p}$, $R_{22}\in {\mathbb{R}}^{q\times q}$ are the symmetric similarity matrices corresponding to the two types of features, respectively, and $R_{12}\in {\mathbb{R}}^{p\times q}$ is the nonnegative similarity matrix between the two types of data. $S_{11}$ and $S_{22}$ represent the matrix describing the similarity of the two networks obtained from the decomposition of $R_{11}$ and $R_{22}$, respectively. Off-diagonal elements in $S_{11}$ and $S_{22}$ indicate the importance of relationships between rois and between genes.

### 2.3. Hypergraph learning

**Figure 1:**
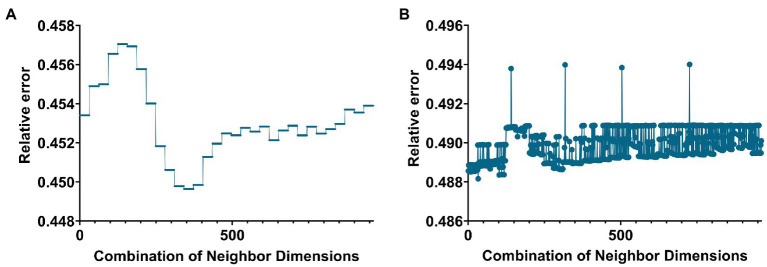
Line graph of the relationship between the number of neighbors and the value of the relative error when building a hypergraph in the two groups. **(A,B)** Are the cases of AD group and MCI group, respectively.

In graph learning, vertices and edges can describe the relationship between multiple objects. However, simple graphs may not depict complex relationships in practical situations. Therefore, hypergraph theory is widely used to mine higher-order correlations between complex things (Xiao et al., 2020). A hypergraph can connect more than two vertices through hyperedges. It is an extension of simple graphs where each edge can connect multiple vertices, known as hyperedges. Let G(V,E,w) represents a hypergraph, and V,E,andw represent vertices, hyperedges, and the weights of vertices, respectively. $V=\left\{v_{1},v_{2},\ldots ,v_{N}\right\}$ is the set of vertices contained in one of the hyperedges $ℰ=\left\{e_{1},e_{2},\ldots ,e_{M}\right\}$ is the set of hyperedges. $w={\left(w\left(e_{1}\right),w\left(e_{2}\right),\ldots ,w\left(e_{M}\right)\right)}^{T}\in {\mathbb{R}}^{M}$ corresponds to the weight of each hyperedge. In addition, the relationship between hyperedges and vertices can be represented by an association matrix H shown on the right side of Figure 1. Among them, the (i,j) element of matrix H indicates whether the j−th hyperedge contains the i−th vertex. The element in the i−th row and j−th column of matrix H is defined as follows:


(3) $$H_{ij}=\{\begin{array}{ll} 1, & \;\mathrm{if}\;v_{i}\in e\\ 0, & \;\mathrm{if}\;v_{i}\notin e \end{array}$$

Among them, $H=\left[H_{ij}\right]\in {\mathbb{R}}^{N\times M}$. Furthermore, the degrees d(v) and δ(e) of the i−th vertex and the j−th hyperedge are defined, respectively.


(4) $$d\left(v_{i}\right)=\sum_{e_{j}\in ℰ}\;w\left(e_{j}\right)H_{ij}\;\text{for}1\leq i\leq N$$

(5) $$\delta \left(e_{j}\right)=\sum_{v_{i}\in V}\;H_{ij}\;\text{for}1\leq j\leq M$$

Further, define degree diagonal matrices $D_{v}$ and $D_{e}$. Among them, $D_{v}=diag\left(d\left(v_{1}\right),d\left(v_{2}\right),\cdots ,d\left(v_{N}\right)\right)\in {\mathbb{R}}^{N\times N}$,$D_{\rho }=diag\left(\delta \left(e_{1}\right),\delta \left(e_{2}\right),\cdots ,\delta \left(e_{M}\right)\right)\in {\mathbb{R}}^{M\times M}$. Furthermore, let W represent the diagonal matrix of hyperedge weights, $W=diag\left(w\right)=diag\left(w\left(e_{1}\right),w\left(e_{2}\right),\cdots ,w\left(e_{M}\right)\right)\in {\mathbb{R}}^{M\times M}$. Then, the similarity matrix S that defines the hypergraph G is as follows.

(6) $$S=HWD_{e}^{-1}H^{T}\in {\mathbb{R}}^{N\times N}$$

Similar to the Laplacian matrix definition for simple graphs, the Laplacian matrix for hypergraphs is defined as follows.


(7) $$L=D_{v}-S$$

### 2.4. Hypergraph-based network non-negative matrix factorization

The previously introduced netNMF algorithm does not consider the correlation within different networks, while the hypergraph Laplacian matrix can integrate high-order correlation information of different modal data. It helps the algorithm identify more biologically meaningful network modules and improves its performance to a certain extent. This paper defines hypergraph regularization as follows.


(8) $$\Omega =G^{T}L^{h}G$$

$L_{1}$ and $L_{2}$ are the hypergraph Laplacian matrices of $X_{1}$ and $X_{2}$, respectively. Further, let $\psi_{1},\psi_{2},\Phi_{1}$, and $\Phi_{2}$ be Lagrangian multipliers, and (9) can be arranged as a Lagrangian function.


(9) $$\begin{array}{c} L=tr\left({\left(R_{11}-G_{1}S_{11}G_{1}^{T}\right)}^{T}\left(R_{11}-G_{1}S_{11}G_{1}^{T}\right)\right)\\ +tr\left({\left(R_{12}-G_{1}G_{2}{}^{T}\right)}^{T}\left(R_{12}-G_{1}G_{2}{}^{T}\right)\right)\\ +tr\left({\left(R_{22}-G_{2}S_{22}G_{2}^{T}\right)}^{T}\left(R_{22}-G_{2}S_{22}G_{2}{}^{T}\right)\right)\\ +\lambda_{1}tr\left(G_{1}^{T}L_{1}G_{1}\right)+\lambda_{2}tr\left(G_{2}{}^{T}L_{2}G_{2}\right)+tr\left(\psi_{1}^{T}S_{11}\right)\\ +tr\left(\psi_{2}^{T}S_{22}\right)+tr\left(\Phi_{1}^{T}G_{1}\right)+tr\left(\Phi_{2}^{T}G_{2}\right) \end{array}$$

The following formula can be obtained using (10) to calculate the partial derivatives of $S_{11}$, $S_{22}$, $G_{1}$ and $G_{2}$, respectively.


(10) $$\begin{array}{l} \frac{\partial f}{\partial S_{II}}=-2G_{1}^{T}R_{11}G_{1}+2G_{1}^{T}G_{1}S_{11}G_{1}^{T}G_{1}+\psi_{l}\\ \frac{\partial f}{\partial S_{22}}=-2G_{2}^{T}R_{22}G_{2}+2G_{2}^{T}G_{2}S_{22}G_{2}^{T}G_{2}+\psi_{2}\\ \frac{\partial f}{\partial G_{1}}=4\left(G_{1}S_{11}G_{1}^{T}G_{1}S_{11}-R_{11}G_{1}S_{11}\right)+2\left(G_{1}G_{2}^{T}G_{2}-R_{12}G_{2}\right)\\ +2\lambda_{1}L_{1}G_{l}+\Phi_{1}\\ \frac{\partial f}{\partial G_{2}}=4\left(G_{2}S_{22}G_{2}^{T}G_{2}S_{22}-R_{22}G_{2}S_{22}\right)+2\left(G_{2}G_{1}^{T}G_{1}-R_{12}G_{1}\right)\\ +2\lambda_{2}L_{2}G_{2}+\Phi_{2} \end{array}$$

Further, through the Karush-Kuhn-Tucher (KKT) condition, the iteration rules of $S_{11}$, $S_{22}$, $G_{1}$ and $G_{2}$ can be obtained, as shown below.


$${\left(s_{11}\right)}_{ij}\leftarrow s_{11}\frac{{\left(G_{1}^{T}R_{11}G_{1}\right)}_{ij}}{{\left(G_{1}^{T}G_{1}S_{11}G_{1}^{T}G_{1}\right)}_{ij}},$$

$${\left(s_{22}\right)}_{ij}\leftarrow s_{22}\frac{{\left(G_{2}^{T}R_{22}G_{2}\right)}_{ij}}{{\left(G_{2}^{T}G_{2}S_{22}G_{2}^{T}G_{2}\right)}_{ij}},$$

$${\left(g_{1}\right)}_{ij}\leftarrow {\left(g_{1}\right)}_{ij}\frac{{\left(R_{12}G_{2}+2R_{11}G_{1}S_{11}\right)}_{ij}}{{\left(2G_{1}S_{11}G_{1}^{T}G_{1}S_{11}+G_{1}G_{2}^{T}G_{2}+2\lambda_{1}L_{1}G_{1}\right)}_{ij}},$$

(11) $${\left(g_{2}\right)}_{ij}\leftarrow {\left(g_{2}\right)}_{ij}\frac{{\left(R_{12}^{T}G_{1}+2R_{22}G_{2}S_{22}\right)}_{ij}}{{\left(\alpha G_{2}G_{1}^{T}G_{1}+2G_{2}S_{22}G_{2}^{T}G_{2}S_{22}+\lambda_{2}L_{2}G_{2}\right)}_{ij.}}$$

### 2.5. Network module selection method

Two types of modules, $G_{1}$ and $G_{2}$, can be identified from the ROI matrix $R_{11}$ and gene network matrix $R_{22}$ constructed in this paper. Specifically, zscore is introduced into this paper to calculate the weights of genes and ROIs, and genes and ROIs with weights above the threshold are treated as members of the corresponding community.


(12) $$x_{ij}=\frac{{\left(G_{l}\right)}_{ij}-u_{\left(G_{l}\right).j}}{\sigma_{\left(G_{l}\right).j}}\;\left(l=1,2\right)$$

Among them, $u_{\left(G_{l}\right).j}=\frac{1}{N}\sum {\left(G_{l}\right)}_{ij}$, $\sigma_{\left(G_{l}\right),j}=\sqrt{\frac{1}{N}\sum {\left({\left(G_{l}\right)}_{ij}-u_{\left(G_{l}\right).j}\right)}^{2}}$. In this paper, the threshold was set to 1.

### 2.6. Evaluation indicators for regression analysis and diagnostic model construction

In this paper, regression analysis and diagnostic model construction were carried out on the Top elements in the network module obtained by the HG-netNMF algorithm. For regression analysis, this paper introduced mean absolute error (MAE) and root mean square error (RMSE) to evaluate the regression performance, and their definitions are as follows:


(13) $$RMSE=\sqrt{\frac{\sum_{i=1}^{n}{\left({\overset{⌢}{y}}_{i}-y_{i}\right)}^{2}}{n}}$$

(14) $$MAE=\frac{\sum_{i=1}^{n}\left|{\overset{⌢}{y}}_{i}-y_{i}\right|}{n}$$

where ${\overset{⌢}{y}}_{i}$ represents the predicted value, and $\;y_{i}$ represents the true value. In addition, when constructing the diagnostic model, this paper introduced the Receiver Operating Characteristic (ROC) curve to evaluate the classification performance of the algorithm, the abscissa is the false positive rate (FPR), and the ordinate is the true positive rate (TPR). The AUC value is the area covered by the ROC curve. AUC can measure the classification effect of the classifier.

## 3. Results

### 3.1. Data acquisition and preprocessing

The real data used in this paper are all from the ADNI database. We collected sMRI imaging and gene expression data from 306 subjects in ADNI. Table 1 gives specific information of the samples.

**Table 1:** Information about the samples included in the analysis in this paper.

Groups	AD	MCI	HC
Number	25	216	65
Gender(Male/Female)	10/15	101/115	31/34
Age(mean ± std)	75.99 ± 10.22	71.56 ± 7.50	75.17 ± 5.86
MCI(mean ± std)	20.48 ± 4.28	28.23 ± 1.74	28.69 ± 1.25

HC stands for Healthy Control.

For sMRI data, this paper realized the segmentation of sMRI based on the CAT toolkit of MATLAB software. Specifically, the CAT toolkit provided a voxel-based morphometric measurement (VBM) function (Veres et al., 2009). Finally, the gray matter volumes of 140 ROIs were extracted as imaging features. Differential expression analysis was performed using the Limma package for gene expression data. Specifically, we used the AD group and the MCI group as the diseased group and the HC group as the control group to experiment. Differentially expressed genes with p values less than 0.01 in the AD and MCI groups were retained (510 genes were retained in the AD group, and 314 genes were retained in the MCI group).

In addition, this paper firstly divided the AD group and the MCI group into the training set and the test set according to the ratio of 8:2 and used the HG-netNMF algorithm to perform network module association analysis on the training set of the two groups of data, and then validate on the test set, and use Top 10 genes to regress Top 10 ROIs, build diagnostic models, etc.

### 3.2. The influence of neighbor size

In this paper, the K-nearest neighbors (KNN) method was used to construct the hyperedge, and the neighbor size of the KNN algorithm needs to be selected. The size of the neighbors was selected based on the size of the objective function value. Based on the selection experience of previous papers, we divided the parameters of the two groups. Figures 1A,B below give the line graphs of the relationship between the neighbor size of the AD group and the MCI group and the objective function value, respectively.

The AD group’s smallest relative error was 0.4496 (corresponding to 31 and 5). The MCI group’s smallest relative error was 0.4848 (corresponding to 5 and 47).

### 3.3. Parameter selection

**Figure 2:**
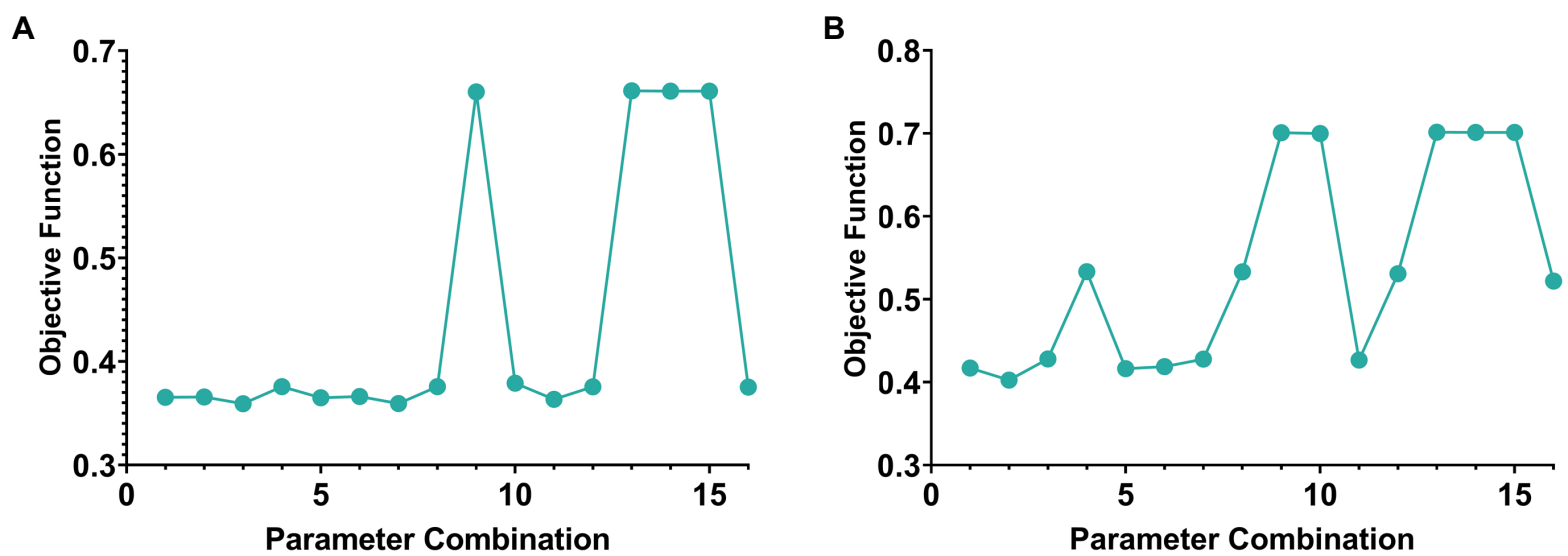
The line graph of parameter selection and objective function value of AD group and MCI group. **(A,B)** Are the cases of AD group and MCI group, respectively.

Two parameters need to be selected in this paper, namely $\lambda_{1}$ and $\lambda_{2}$. The objective function value was selected as the selection standard, and parameters were selected from the range of [0.0001, 0.001, 0.01, 0.1]. The schematic diagram of parameter selection was shown in Figure 2.

In addition, according to the selection experience of previous literature, the value of k generally does not exceed one-tenth of the minimum number of samples or features for the number of genes-ROIs network modules k. Therefore, the k value in this paper was set to 7 in the AD group and 22 in the MCI group.

### 3.4. Algorithm comparison

To confirm the superiority of the algorithm, the algorithm was compared on simulated data and the real data. First, on simulated data, this paper compared the objective function values of the two algorithms at different noise levels. The generation method of simulated data was similar to Kim et al. (2019). Specifically, this paper simulated the sMRI numerical matrix X and the gene numerical matrix Y of 300 random samples. By defining a normal distribution $N\left(0,\sigma_{\epsilon }^{2}\right)$, generate an ROI weight vector u with 200 elements and a gene weight vector v with 2000 elements. In addition, this paper created noise ϵ=e, which comes from a normal distribution $N\left(0,\sigma_{e}^{2}\right)$. Next, correlated and uncorrelated ROI and gene variables were generated similarly: X=uε+e and X=e and Y=vε+e and Y=e. The respective and mutual Pearson correlation coefficients were then calculated for the two variables as inputs to the algorithm ($R_{11}$, $R_{22}$, and $R_{12}$). Next, the objective function values of the proposed method and the netNMF algorithm were compared under the same experimental conditions and noise levels (Table 2). In addition, we simulated the anti-noise performance of the two algorithms when the sample size is large. Specifically, we set the total number of samples to 1,000, and the remaining parameters are consistent with the above to obtain the objective function values of the two algorithms under different noise levels (Table 3).


$$R_{11}=G_{1}S_{11}G_{1}^{T}$$

$$R_{12}=G_{1}G_{2}{}^{T}$$

(15) $$R_{22}=G_{2}S_{22}G_{2}^{T}$$

**Table 2:** The objective function values of the two algorithms were compared on a simulation dataset with a small sample size.

Noise Level	1	2	3	4	5
HG-netNMF	**0.3495**	**0.5909**	**0.7200**	**0.7902**	**0.8179**
netNMF	0.3500	0.5919	0.7204	0.7908	0.8185

The values in bold indicate that the algorithm has a smaller objective function value at this noise level.

**Table 3:** The objective function values of the two algorithms were compared on a simulation dataset with a large sample size.

Noise level	1	2	3	4	5
HG-netNMF	**0.2864**	**0.5162**	**0.6769**	**0.7720**	**0.8281**
netNMF	0.2867	0.5167	0.6796	0.7731	0.8315

The values in bold indicate that the algorithm has a smaller objective function value at this noise level.

In real data, this paper compared the results of the proposed HG-netNMF algorithm with the results obtained by the netNMF algorithm for the AD group and the MCI group. Specifically, we calculated the Pearson correlation coefficients between the reconstructed matrices $R_{11}^{\prime }$, $R_{22}^{\prime }$, and $R_{12}^{\prime }$ and the three original matrices $R_{11}$, $R_{22}$, and $R_{12}$, as shown in Table 4. In addition, we give the formulas for $R_{11}^{\prime }$, $R_{22}^{\prime }$ and $R_{12}^{\prime }$ in the following formula.

**Table 4:** Pearson correlation coefficients between the three original matrices and the three reconstructed matrices obtained by the two algorithms in the AD group and the MCI group.

	netNMF(AD)	HG-netNMF(AD)	NetNMF(MCI)	HG-netNMF(MCI)
$corr\left(R_{11},R_{11}^{\prime }\right)$	**0.9493**	0.9427	0.9500	**0.9559**
$corr\left(R_{22},R_{22}^{\prime }\right)$	0.8877	**0.8929**	0.8532	**0.8542**
$corr\left(R_{12},R_{12}^{\prime }\right)$	0.8871	**0.8923**	0.9030	**0.9136**

corr(X,Y) represents the Pearson correlation coefficient between X and Y. It can be seen from Table 3 that the proposed algorithm outperforms the netNMF algorithm in the reconstruction of $R_{22}$ and $R_{12}$ in the AD group. In the MCI group, the proposed algorithm outperformed the netNMF algorithm for reconstructing the three original matrices. This confirmed that hypergraph regularization contributes to the improvement of algorithm reconstruction performance. In addition, this paper also drew the scatter plots of the two algorithms between the three original matrices and the three reconstructed matrices in the AD group and the MCI group, as shown in Figure 3.

**Figure 3:**
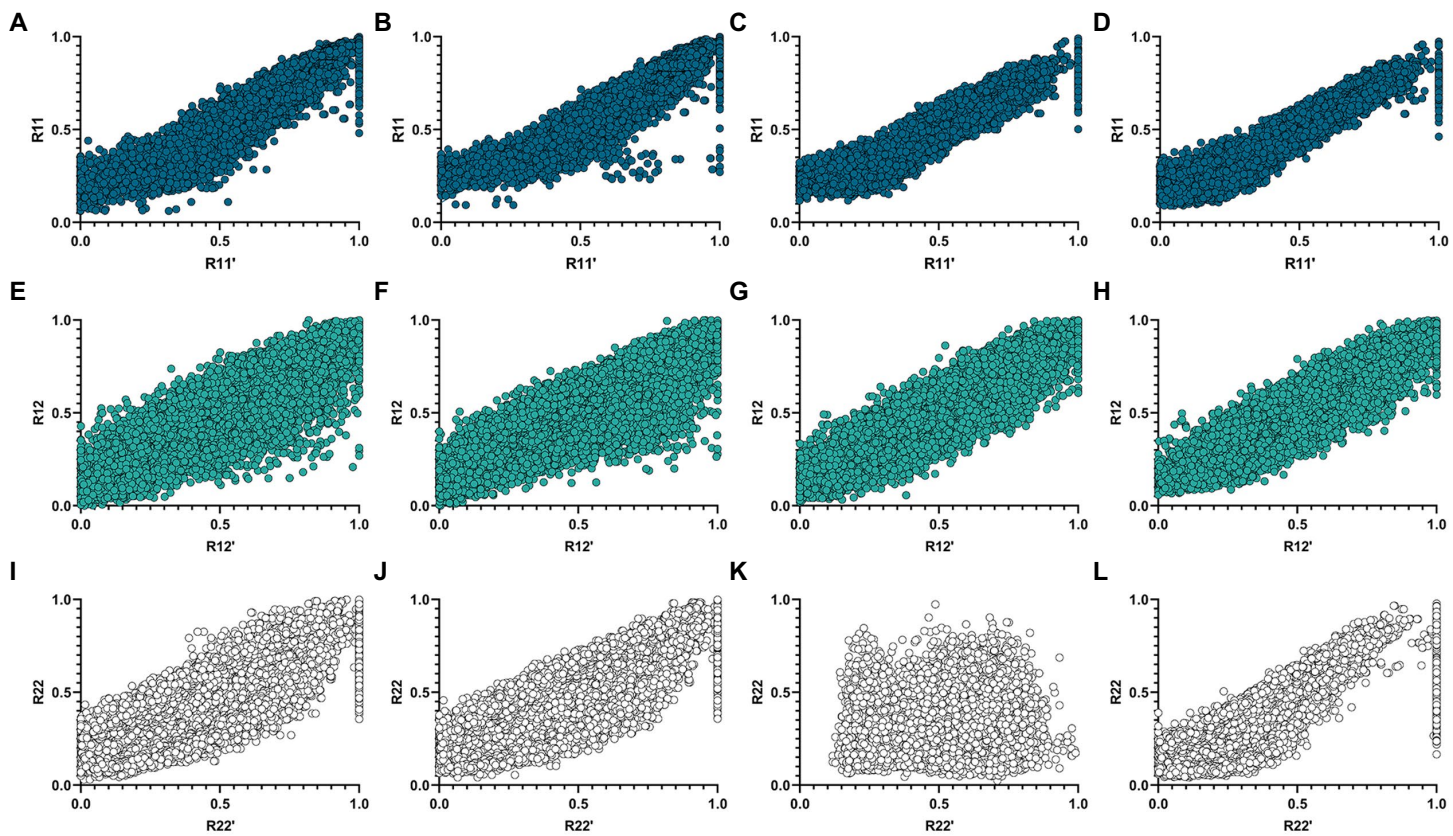
Correlation scatter plots of $R_{11}$, $R_{12}$, and $R_{22}$ of the two algorithms and their reconstruction matrices $R_{11}^{\prime }$, $R_{12}^{\prime }$, and $R_{22}^{\prime }$ in the AD and MCI groups. **(A,E,I)** Are three sets of scatter plots obtained by the netNMF algorithm in the AD group. **(B,F,J)** Are three groups of scatter plots obtained by the HG-netNMF algorithm in the AD group. **(C,G,K)** Are three sets of scatter plots obtained by the netNMF algorithm in the MCI group. **(D,H,L)** Are three groups of scatter plots obtained by the HG-netNMF algorithm in the MCI group.

As seen from Figure 3, the two algorithms had comparable reconstruction performance when reconstructing $R_{11}$ and $R_{12}$. However, the proposed algorithm performed better when reconstructing $R_{22}$ in the MCI group.

### 3.5. Network module selection

**Figure 4:**
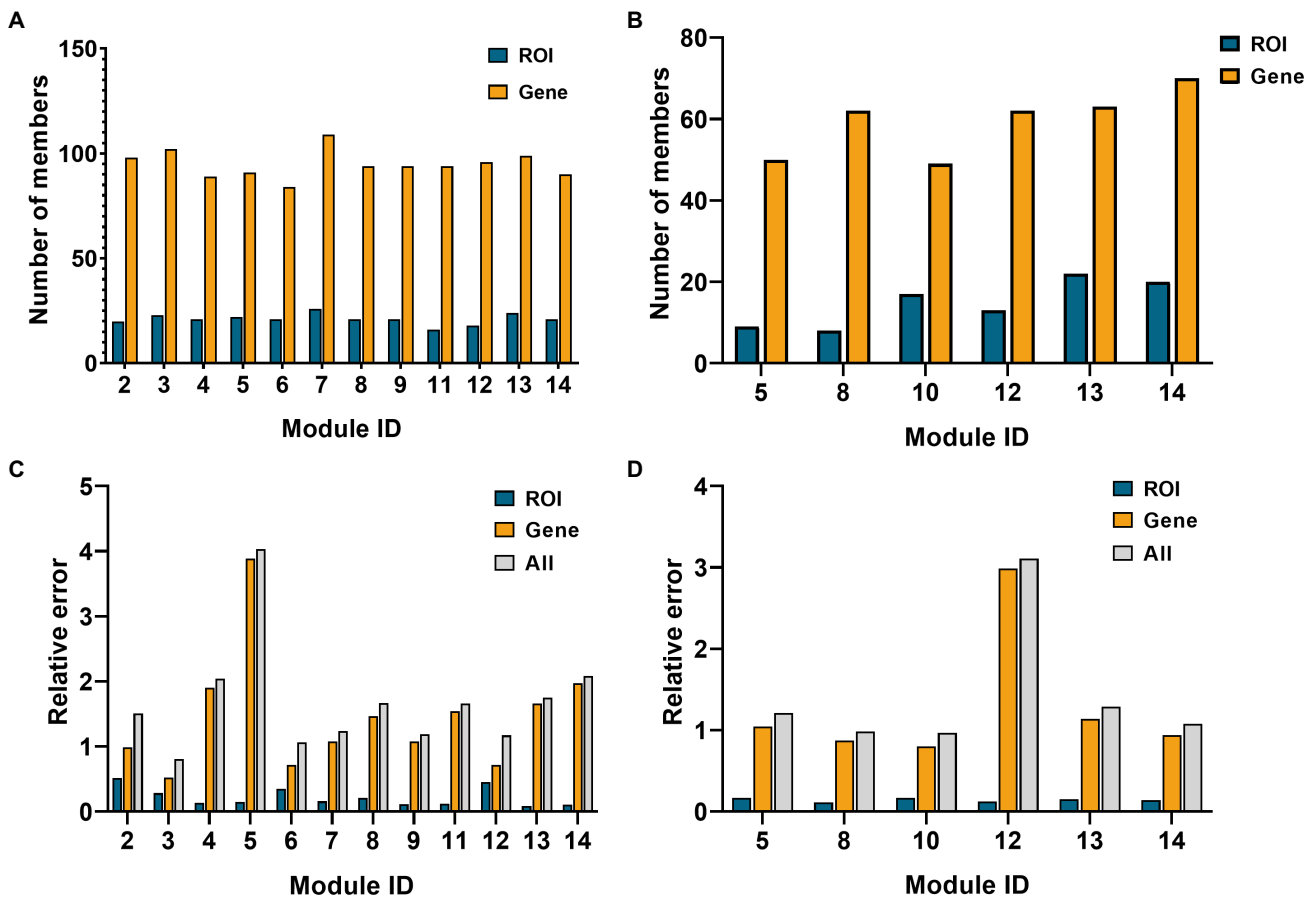
Histogram of network module selection in the AD group and MCI group. **(A,B)** Are the element numbers of ROIs and genes in the retained modules in the AD and MCI groups, respectively. **(C,D)** Are the ROIs, genes, and relative errors of the two in the retained modules in AD and MCI groups, respectively.

Fourteen network modules were obtained in the AD group, of which module 1 and 10 contained less than five genes, so they were eliminated. In the MCI group, 14 modules were obtained, of which modules 1, 3, 6, 9, and 11 contained less than five ROIs, and modules 2, 3, 4, 7, and 9 contained less than five genes. Therefore, the above modules were eliminated. Figure 4 below shows the number of elements retained in the AD and the MCI group and the respective reconstruction errors and total reconstruction errors of the two modal features.

As shown in Figure 4, module 3 of the AD group and module 10 of the MCI group had minor relative errors, and subsequent analysis of these two modules will be performed later.

### 3.6. Significant network module analysis

**Figure 5:**
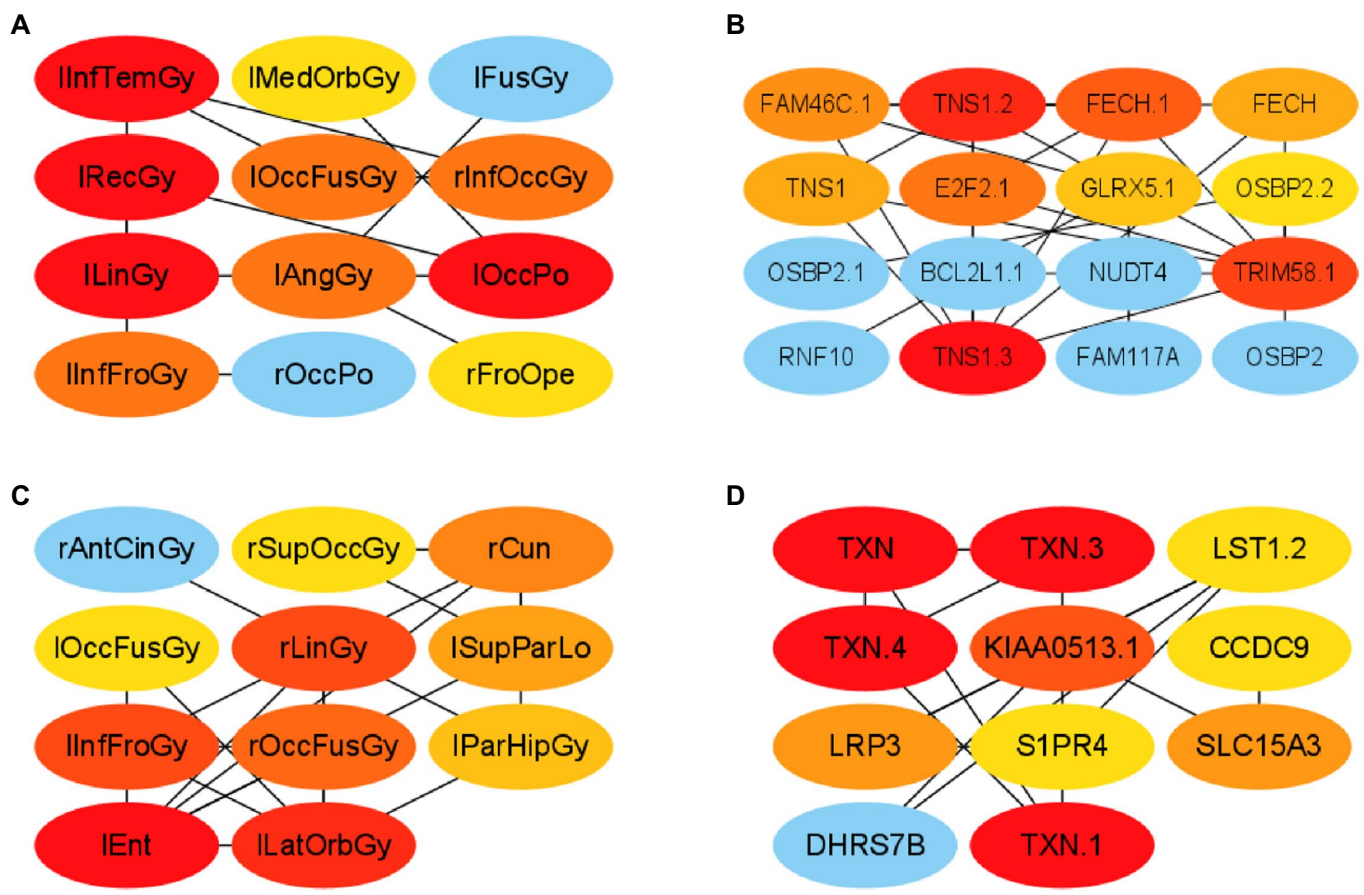
Visualization of ROI-ROI and gene–gene interaction networks in the AD and MCI groups. **(A,B)** are the network diagrams of the ROIs and genes of the AD group, respectively. **(C**,**D)** Are the network maps of the ROIs and genes of the MCI group, respectively.

First, this paper constructed a PPI network using the ROIs and genes in network module 3 in the AD group, respectively. Specifically, this paper selected elements in module 3 from $R_{11}$ and $R_{22}$ to form PCC pairs with a one-to-one correspondence between two components. The AD group’s mean of the PCC of gene–gene and ROI-ROI was 0.902. Therefore, in this paper, the PCC threshold of ROI and genes was set to 0.9, and uses the relationship pair of PCC > 0.9 to construct the ROI-ROI interaction network and the gene–gene interaction network. Furthermore, the interaction network model was visualized using Cytoscape (version 3.9.1; Shannon et al., 2003). The Matthews Correlation Coefficient metric (MCC) algorithm (Boughorbel et al., 2017) was widely used as a performance metric in bioinformatics. This paper used the MCC algorithm to mine the scores of ROIs and genes related to other network nodes and then arranges the top 10 ROIs and genes as the risk ROI and key genes. The MCI group’s mean of the PCC of gene–gene and ROI-ROI was 0.604. Therefore, in this paper, the PCC threshold of ROI and genes was set to 0.60, and used the relationship pair of PCC > 0.6 to construct the ROI-ROI interaction network and the gene–gene interaction network. The PPI networks of the two groups are shown in Figure 5.

### 3.7. Key regions of interests and genes selection

**Figure 6:**
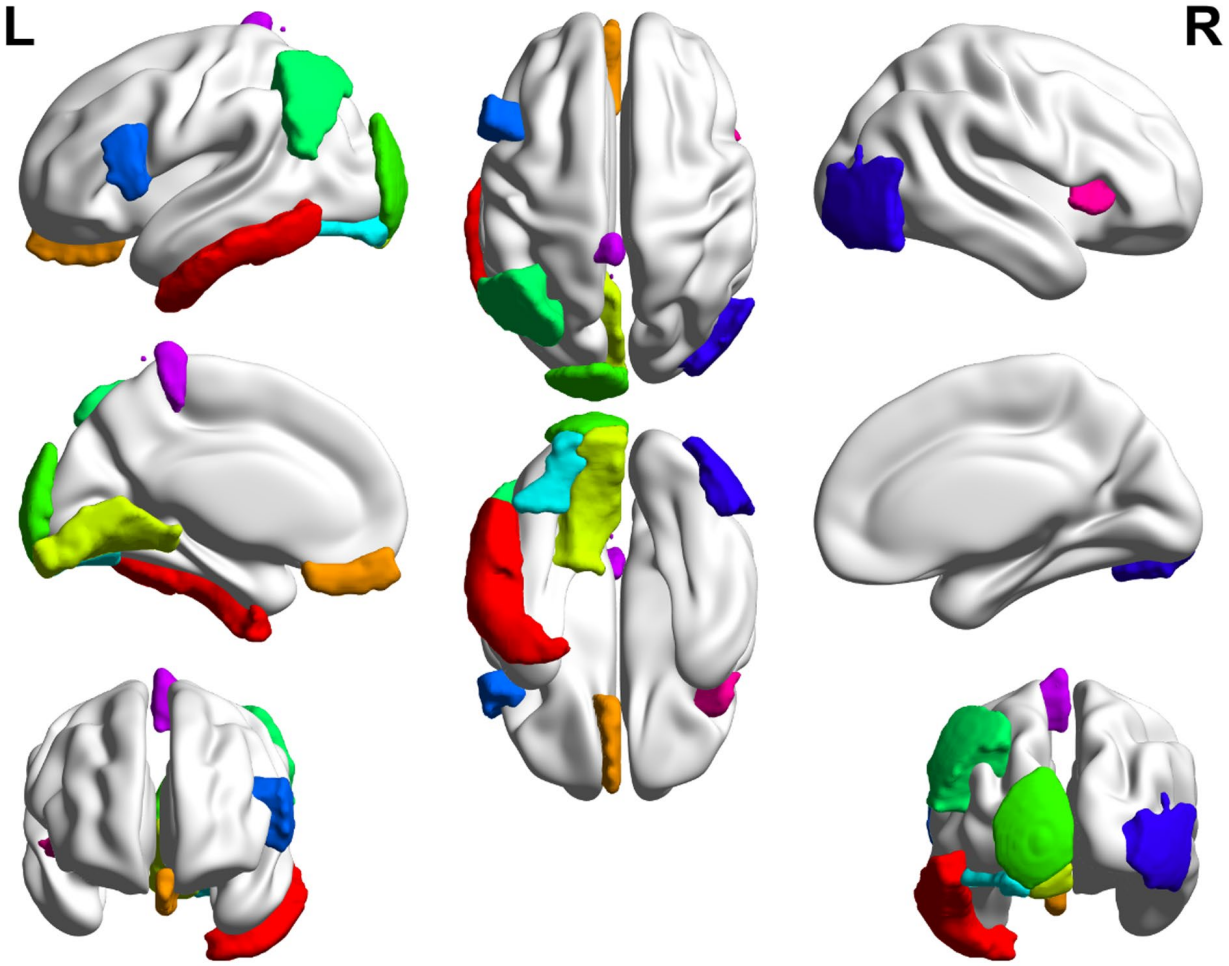
Top 10 ROI visualization of AD group.

**Figure 7:**
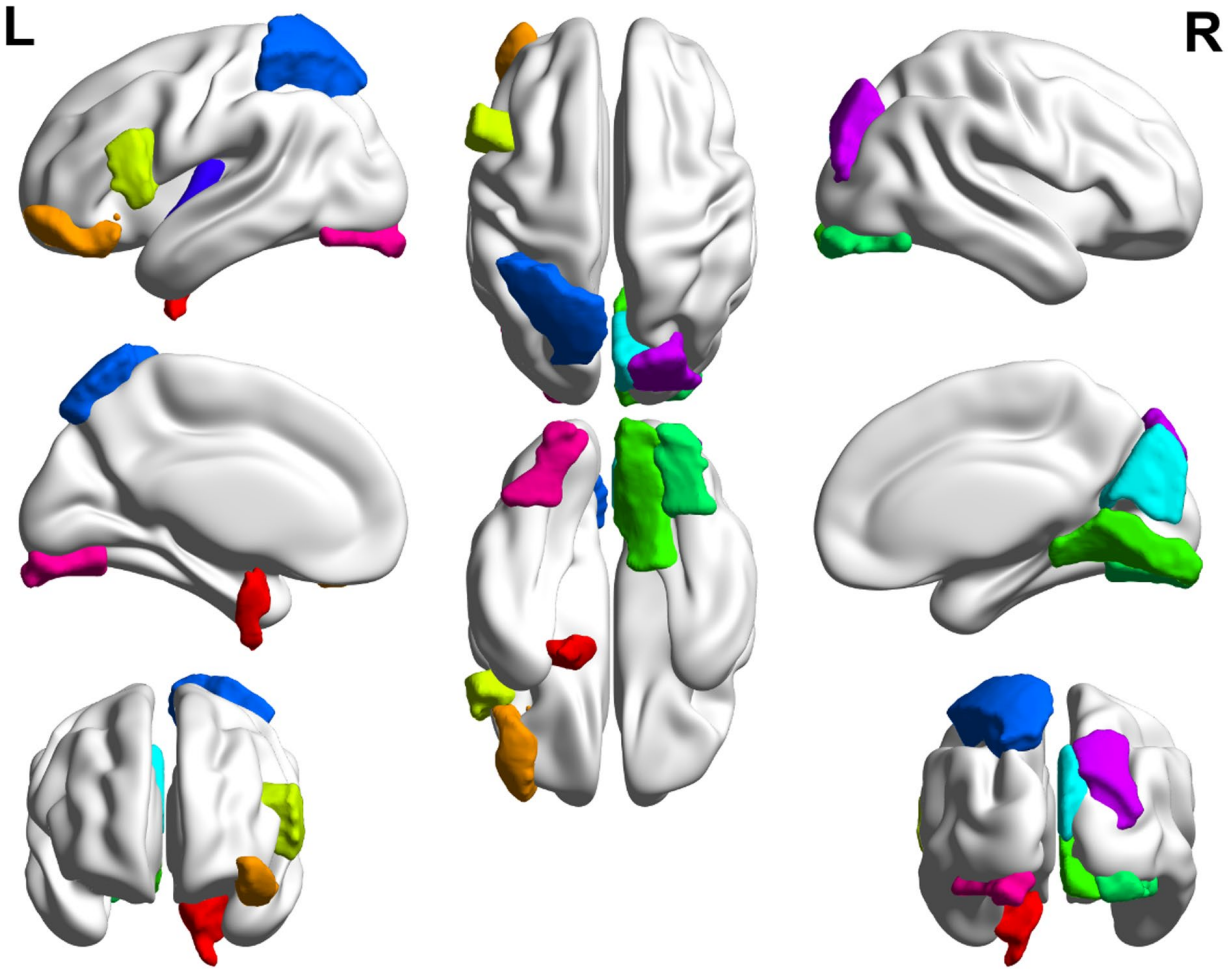
Top 10 ROI visualization of MCI group.

**Figure 8:**
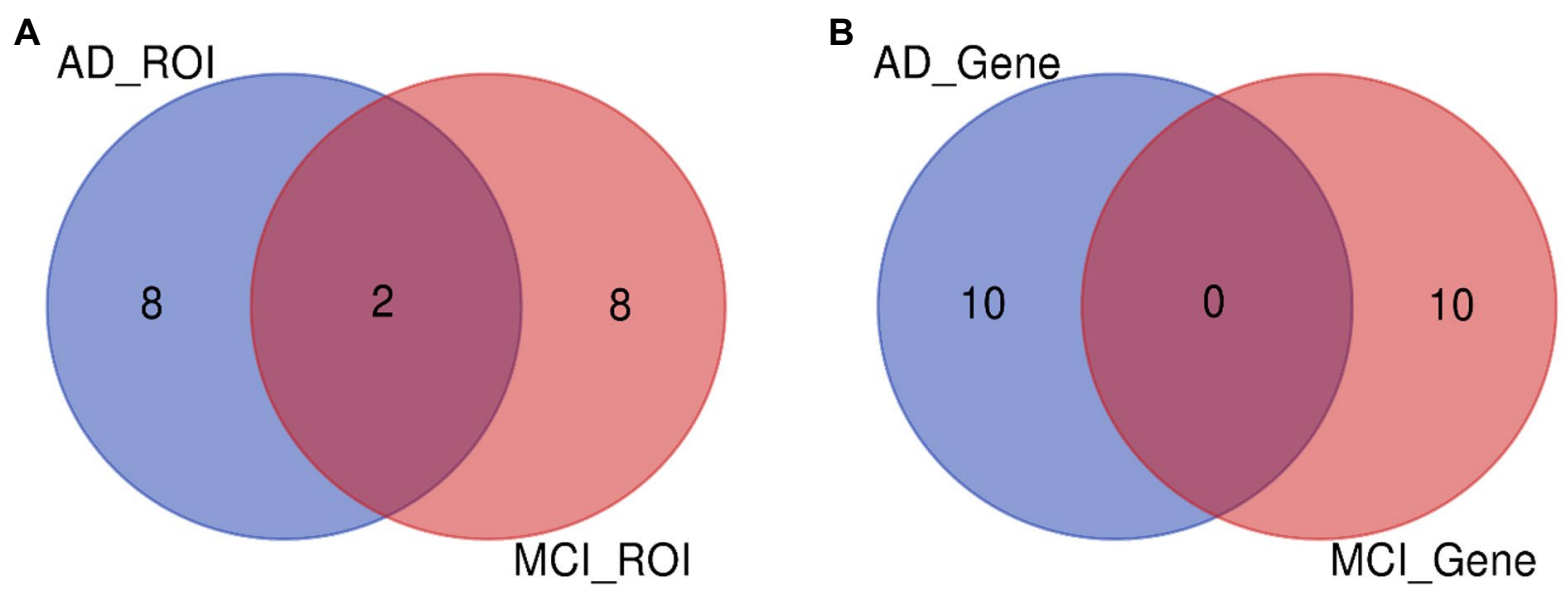
ROI and gene intersection of Top 10 in AD and MCI groups. **(A)** Is the intersection of ROI selected by AD group and MCI group. **(B)** Is the intersection of ROI selected by AD group and MCI group.

In this paper, the MCC algorithm was used to obtain the scores of the Top nodes in the four network graphs of the AD and the MCI groups, as shown in Tables 5, 6. A higher score in the table represents a more critical role for this ROIs/gene in MCI/AD. So we discussed these ROIs/genes in detail in the subsequent discussion section. The top brain regions in both groups were also visualized (Figure 6, Figure 7). We also drew Venn diagrams for ROI and gene nodes in the AD group and MCI group network (Figure 8).

**Table 5:** Top 10 ROIs and genes and their MCC score information in AD group.

ROI	MCC score	Gene	MCC score
Left Inferior Temporal Gyrus	3	TNS1.3	44
Left Gyrus Rectus	3	TNS1.2	42
Left Lingual Gyrus	3	TRIM58.1	39
Left Occipital Pole	3	FECH.1	30
Left Angular Gyrus	2	E2F2.1	24
Left Occipital Fusiform Gyrus	2	FAM46C.1	7
Left Inferior Frontal Gyrus	2	FECH	6
Right Inferior Occipital Gyrus	2	TNS1	6
Left Medial Orbital Gyrus	1	GLRX5.1	4
Right Frontal Operculum	1	BCL2L1.1	3

**Table 6:** Top 10 ROIs and genes and their MCC score information in MCI group.

ROI	MCC score	Gene	MCC score
Left Entorhinal Area	28	TXN.3	6
Left Lateral Orbital Gyrus	27	TXN.4	6
Left Inferior Frontal Gyrus	26	TXN.1	6
Right Lingual Gyrus	26	TXN	6
Right Occipital Fusiform Gyrus	24	KIAA0513.1	4
Right Cuneus	7	SLC15A3	2
Left Superior Parietal Lobule	4	DHRS7B	2
Left Para hippocampus Gyrus	3	LST1.2	2
Right Superior Occipital Gyrus	2	CCDC9	1
Left Occipital Fusiform Gyrus	2	LRP3	1

## 4. Discussion

### 4.1. Analysis of the biological significance of key regions of interests and genes

We first analyzed the biological significance of the Top 10 ROIs in the AD group. A pilot study showed that in AD, Aβ deposition in the inferior temporal gyrus was strongly associated with gray matter atrophy in brain region BF-227 (Maeno, 2019). The left lingual gyrus is associated with changes in functional connectivity at the local network level in AD (Chang et al., 2020). The left angular gyrus is associated with increased brain metabolism in AD patients (Weissberger et al., 2017). Studies on rs-fMRI have shown that the left angular gyrus is the brain functional connectivity region showing the most significant discrimination (Wang et al., 2021). The left inferior frontal gyrus is associated with genetic variants of cortical atrophy in AD, contributing to further understanding AD’s genetic basis (Kim et al., 2021). Maeno (2019) found that Aβ deposition in the anterior cuneiform of AD patients was associated with atrophy of the right occipitotemporal region. The left medial orbital age has a strong negative correlation, which is valid in the age adjustment of AD subjects (Wang et al., 2021).

We also analyzed the Top ROIs in the MCI group’s biological significance. The left entorhinal cortex decreases in volume and cognitive and motor dysfunction in older adults with mild cognitive impairment (Sakurai et al., 2019). Cerebral blood flow (rCBF) in the left lateral orbital gyrus was decreased in the dizzy MCI group compared with the non-dizzy MCI group (Na et al., 2020). In an ALFF-based fMRI study, researchers found significantly increased ALFF values in the right lingual gyrus of MCI patients compared with HC (Lai et al., 2022). Occipital gray matter volume correlates with neuropsychological performance in patients with amnestic MCI or mild AD (Arlt et al., 2013). The left superior parietal lobule is associated with modulating the amplitude of low-frequency fluctuations (ALFF) in patients with mild cognitive impairment (MCI) (Zhuang et al., 2020). Chen et al. (2020) found atrophy of the Para hippocampus gyrus structure in MCI. In addition, it can be found from Tables 4, 5 that the left inferior frontal gyrus and left inferior frontal gyrus are the intersection ROIs of the two groups. Here we found that these two brain regions were selected in both the AD and MCI groups. Using the left inferior frontal gyrus as a seed, Pistono et al. (2021) found that the functional connectivity of the language network could better discriminate the MCI and AD participants than the executive control network, revealing an increase in connectivity during the MCI phase. To study the relationship between MCI patients and apathy, the researchers divided MCI patients into those with and without “SPECT images suggestive of AD.” They found that apathy negatively correlated with regional cerebral blood flow in the bilateral fusiform gyrus (Kazui et al., 2016).

Next, this paper also performed the same analysis on the Top 10 genes. Two significant features of AD are transcriptome dysregulation and altered RNA-binding protein (RBP) function (Chen et al., 2020). TNS1-related Gene Ontology annotations included RNA binding and actin binding. Variations in actin-involved endocytosis pathways are significant contributors to the overall regulation of genetic risk for AD (Pistono et al., 2021). TRIM58 gene induces E3 ubiquitin ligase in late erythropoiesis (Kazui et al., 2016). Experiments have confirmed that α-synuclein in red blood cells may help differentiate AD from HC. Erythropoiesis is associated with FECH. The protein encoded by FECH is localized to mitochondria (Kazui et al., 2016). GLRX5 encodes a mitochondrial protein (Rybak-Wolf and Plass, 2021). E2F2 is involved in the control of the cell cycle, and Zhou et al. (2021) identified vital cell cycle regulators, helping to develop potential pathways for optimal AD treatment. BCL2L1 was identified as a core target of tau pathogenesis (Tesi et al., 2020).

TXN is associated with cellular senescence, and Gene Ontology annotations associated with this gene include RNA binding and oxidoreductase activity. Chico et al. (2013) found that superoxide dismutase activity was reduced in MCI compared to controls. SLC15A3 is involved in the innate immune response, and Fiala et al. (2017) found that the innate immune system in MCI patients is highly increased or decreased through the transcription of inflammatory genes. LST1 may regulate immune responses, and peripheral innate immune responses had the highest activation level in the MCI group compared with the subjective memory complaints (SMC) and AD groups (Munawara et al., 2021). The CCDC9 gene is a possible component of the exon junction complex (EJC), which is involved in mRNA translation, one of the common pathways of MCI (Fernández-Martínez et al., 2020). LRP3 is involved in regulating gene expression, and analysis of gene expression data from blood may help differentiate MCI from AD (Bottero and Potashkin, 2019).

### 4.2. Analysis of MMSE based on top features

**Figure 9:**
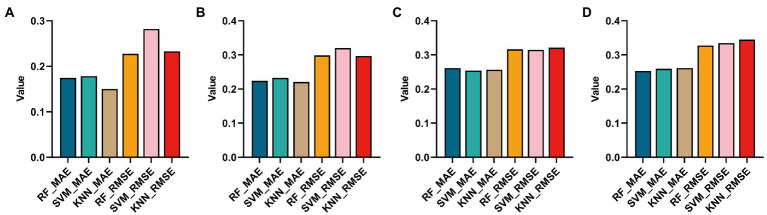
Histograms obtained by using three regression methods (RF, SVM and KNN) to regress MMSE and using two regression evaluation indicators (MAE and MSE). **(A,B)** Are the regression results using the Top ROIs and genes of the AD group, respectively. **(C,D)** Are the regression results of Top ROIs genes using the MCI group, respectively.

In this paper, the Top 10 ROIs and 10 genes of the AD and the MCI groups were used to predict MMSE (stands for root mean square error) to confirm the correlation between the Top features and the clinical score. Specifically, this paper first used support vector regression (SVR), random forest (RF), and K-Nearest Neighbors (KNN) algorithms to regress the MMSE of the two groups on the training sets of the two groups, respectively. The regression effect was evaluated using MAE and RMSE, as shown in Figure 9.

It can be found from Figures 9A,B that using KNN regression algorithm in AD group can get smaller MAE and RMSE in most cases. From Figures 9C,D, it can be found that using the RF algorithm in AD group can get smaller MAE and RMSE in most cases.

### 4.3. Correlation analysis between top regions of interests and genes

**Figure 10:**
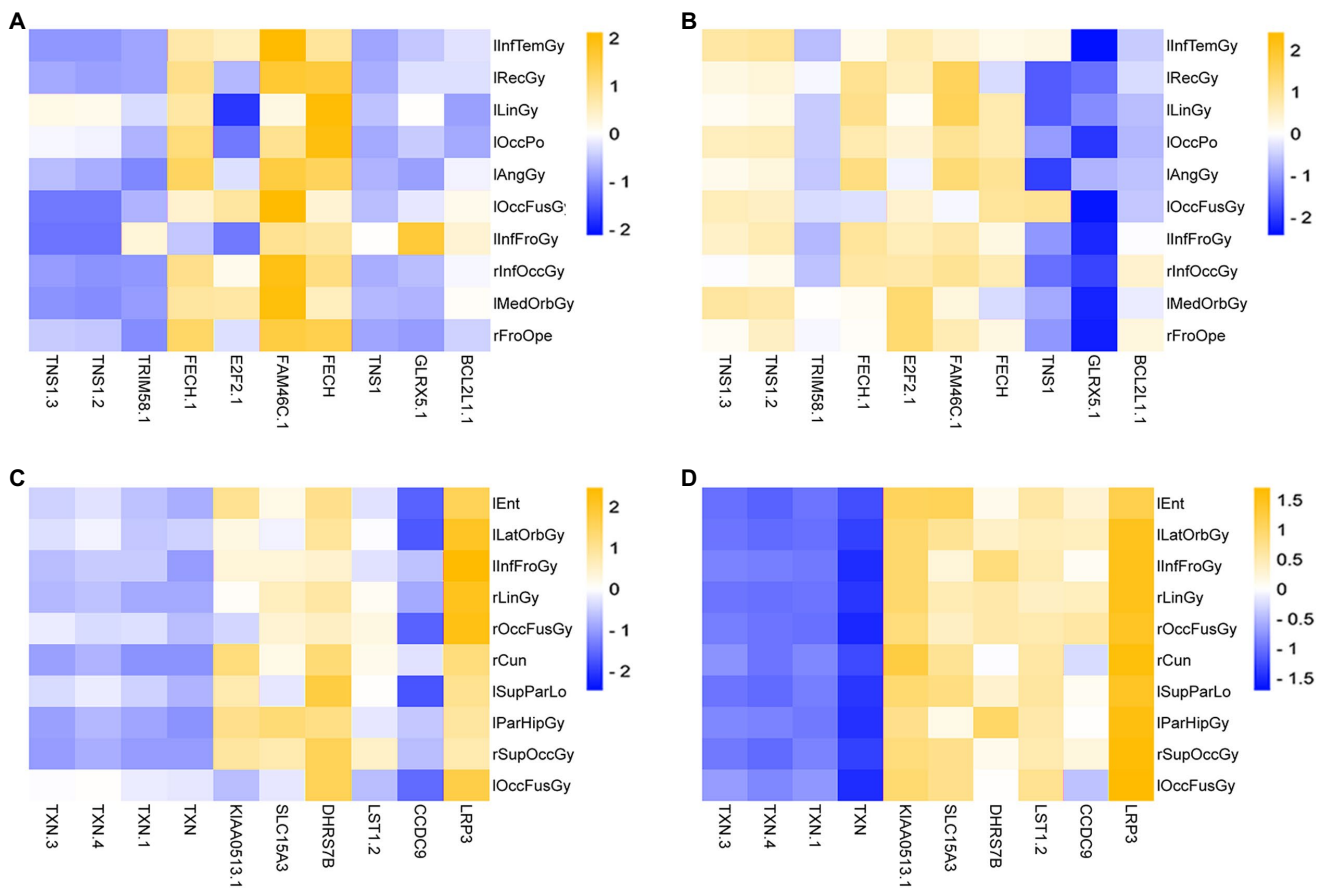
Heatmap of the correlation of Top ROIs and genes on the test set. **(A,B)** Are the correlation heatmaps of AD group and healthy control group, respectively. **(C,D)** Are the correlation heatmaps of the MCI group and the healthy control group, respectively.

This paper drew a correlation heat map for the Top 10 ROIs and genes on the test set in the two groups obtained above, as shown in Figure 10.

As seen in Figure 10, there is a strong correlation between the Top features of the AD group data. This paper takes the absolute value of PCC and then calculates the average correlation. Among them, the average correlation for the AD group was 0.5411, and the correlation of the control group was 0.1045. The mean correlation was 0.1039 in the MCI group and 0.2619 in the control group.

### 4.4. Regression analysis between top features

**Figure 11:**
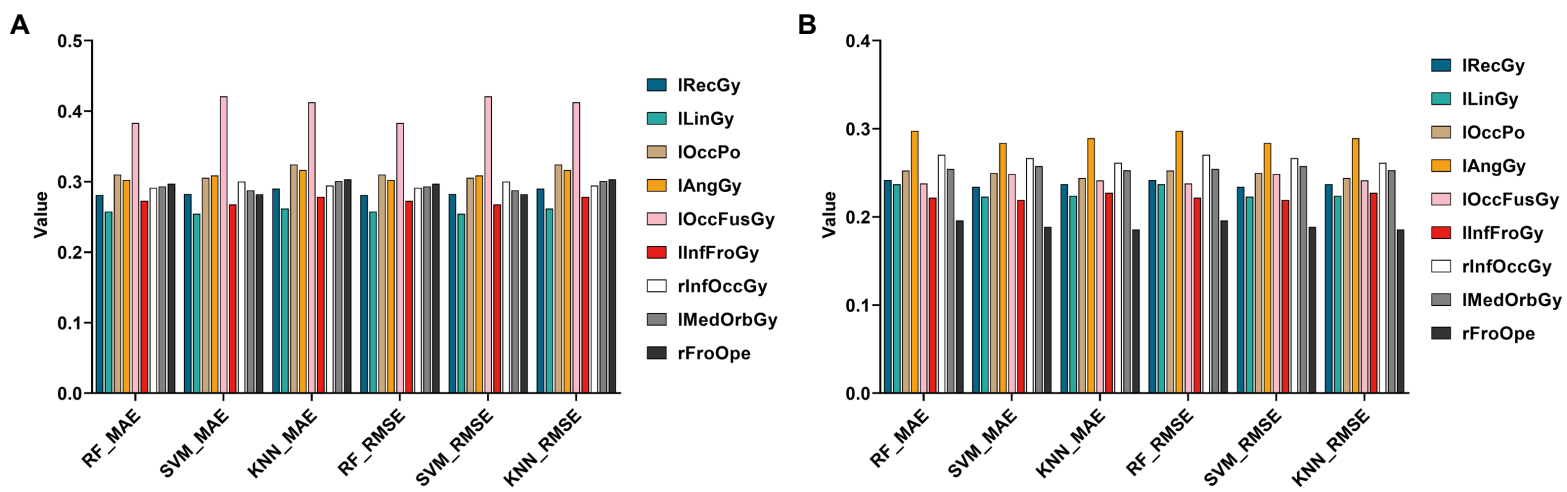
Histograms of errors (MAE and RMSE) obtained by regression prediction of Top 10 ROIs using Top10 genes in the AD and MCI groups, respectively. **(A,B)** Are the regression results of two groups, respectively.

In this section, this paper still used three regression algorithms: SVR, RF, and KNN. Specifically, this paper uses the Top 10 genes selected by the HG-netNMF algorithm to regress to the Top 10 ROIs, respectively, trains three models on the training set, and uses MAE and RMSE to evaluate the regression effect on the test set, as shown in Figure 11.

It can be found from Figure 11 that using different regression algorithms to predict the left lingual gyrus of the AD group can achieve the smallest root mean square error. Using different regression algorithms to predict the right frontal operculum of the MCI group can achieve the smallest root mean square error.

### 4.5. Classification analysis

**Figure 12:**
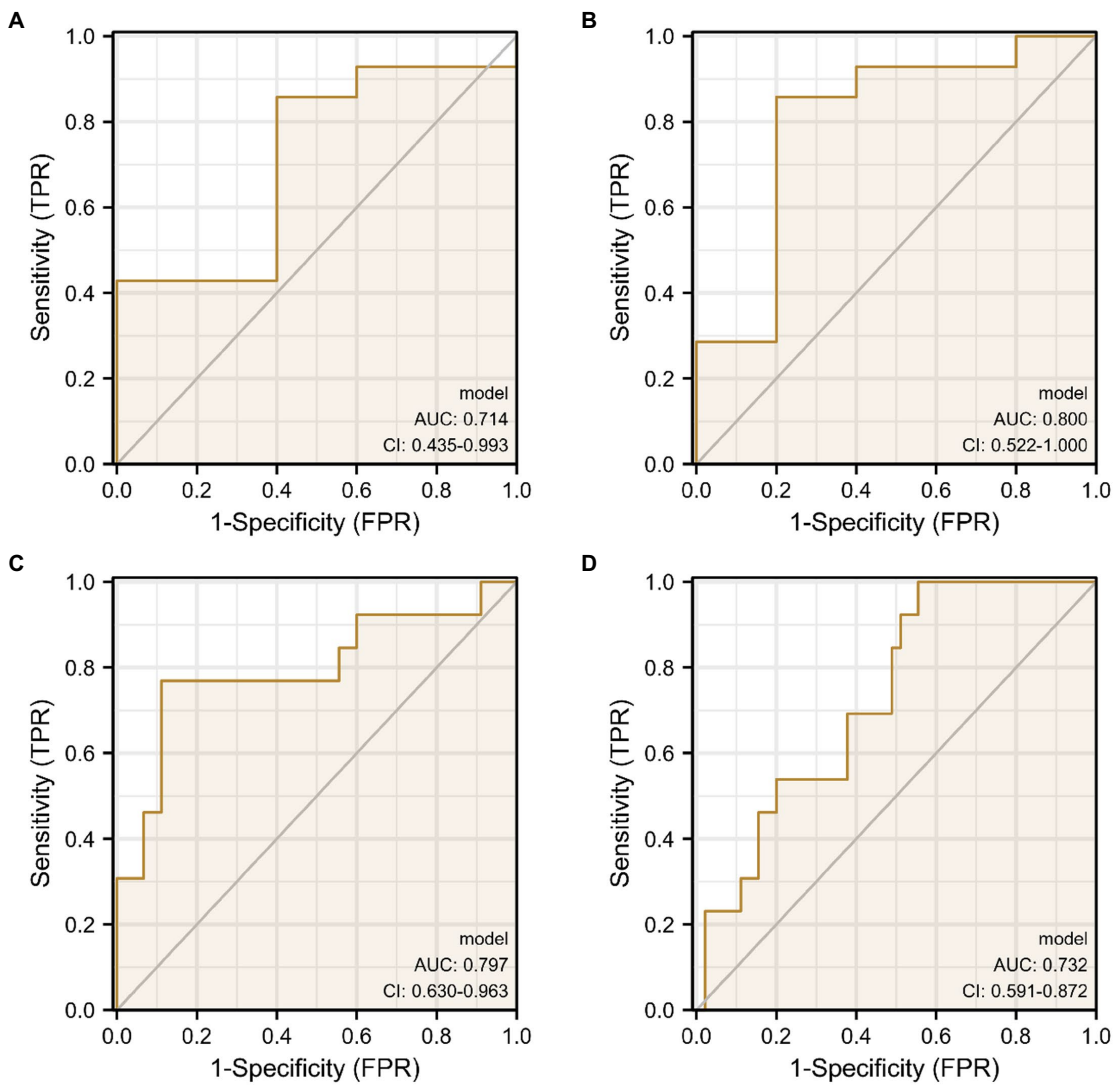
ROC curves obtained from the diagnostic models of AD and MCI constructed using Top ROIs and genes of AD and MCI groups, respectively. **(A,B)** Are the diagnostic models of AD constructed using the Top features of the AD group, respectively. **(C,D)** Are the diagnostic models of MCI constructed using the Top features of the MCI group, respectively.

This paper’s ROC curves were drawn using the Top 10 ROIs and genes of the AD and MCI groups, respectively. In addition, this paper used Logistic regression in IBM SPSS Statistics software to build a joint diagnostic model, as shown in Figure 12. This paper also counted the specific information of the four diagnostic models, as shown in Table 7.

**Table 7:** Specific information of ROC curve.

	AUC	Confidence interval	Sensitivity	specificity
Figure 9A	0.714	0.435–0.993	0.857	0.6
Figure 9B	0.8	0.522–1.000	0.857	0.8
Figure 9C	0.797	0.63–0.963	0.769	0.889
Figure 9D	0.732	0.59–0.872	1	0.444

Next, to determine whether the ROIs and genes involved in the construction of the diagnostic model also have diagnostic significance for AD and MCI, this paper drew ROC curves for the ROIs and genes involved in the construction of the diagnostic model in Figure 9 to verify one by one, as shown in Figure 10. In addition, the ROC curve information of a single ROI and gene in the two groups was calculated separately, as shown in Tables 8, 9.

**Table 8:** ROC curve information of a single top features in AD group.

	AUC	Confidence interval	Sensitivity	specificity
lInfTemGy	0.7	0.441–0.959	0.714	0.8
lRecGy	0.771	0.554–0.989	0.786	0.8
lLinGy	0.786	0.577–0.994	0.714	1
lOccPo	0.743	0.519–0.966	0.643	1
lAngGy	0.771	0.556–0.987	0.714	1
lOccFusGy	0.686	0.426–0.946	0.643	0.8
lInfFroGy	0.743	0.513–0.973	0.643	1
rInfOccGy	0.686	0.393–0.979	0.714	0.8
lMedOrbGy	0.657	0.341–0.973	0.786	0.8
rFroOpe	0.671	0.382–0.961	0.857	0.6
TNS1.3	0.643	0.286–1	1	0.4
TNS1.2	0.586	0.236–0.935	0.714	0.6
FECH.1	0.743	0.488–0.997	0.857	0.6
E2F2.1	0.657	0.302–1	0.929	0.6
FAM46C.1	0.743	0.421–1	0.786	0.8
FECH	0.571	0.229–0.914	0.571	0.8
TNS1	0.693	0.353–1	0.714	0.8
GLRX5.1	0.571	0.191–0.952	1	0.4
BCL2L1.1	0.657	0.407–0.908	0.643	0.8

**Table 9:** ROC curve information of a single top features in the MCI group.

	AUC	Confidence interval	Sensitivity	specificity
lEnt	0.591	0.409–0.774	0.846	0.422
lLatOrbGy	0.520	0.333–0.706	0.692	0.511
rLinGy	0.523	0.336–0.71	0.923	0.2
rCun	0.573	0.378–0.768	0.385	0.889
lSupParLo	0.545	0.353–0.738	0.231	0.911
lParHipGy	0.576	0.384–0.768	0.615	0.689
rSupOccGy	0.537	0.346–0.727	0.538	0.622
lOccFusGy	0.583	0.382–0.784	0.385	0.889
TXN.4	0.552	0.369–0.735	1	0.178
KIAA0513.1	0.578	0.390–0.765	0.538	0.711
DHRS7B	0.61	0.426–0.794	0.615	0.711
LST1.2	0.520	0.313–0.727	0.462	0.733
CCDC9	0.651	0.464–0.838	0.769	0.644
LRP3	0.547	0.36–0.734	0.231	0.933

**Figure 13:**
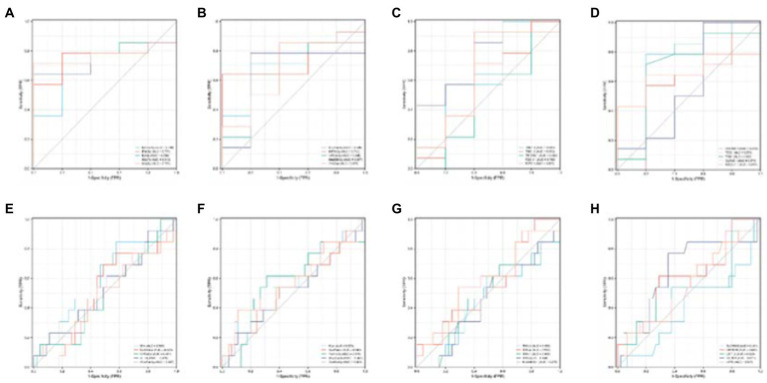
Single validation of Top 10 ROIs and genes for the two groups. **(A–D)** Are the ROC curves of the first five and last five ROIs of the AD group, respectively. **(E–H)** Are the ROC curves of the first five and last five ROIs of the MCI group.

It can be seen from Figure 13 that most of the AUCs of ROI/gene involved in the construction of the diagnostic model are more significant than 0.5, which has diagnostic significance for AD and MCI, further confirming the effectiveness of the algorithm.

## 5. Conclusion

This paper proposed a hypergraph-based NetNMF method to integrate sMRI and genetic data of AD and MCI patients and mined their respective regulatory networks and interaction information, aiming to explore the risk ROIs and genes associated with AD and MCI. Finally, robust diagnostic models for AD and MCI were constructed, respectively. In future work, we will integrate more data types to analyze the disease-related regulatory mechanisms more comprehensively.

## Data availability statement

Publicly available datasets were analyzed in this study. This data can be found at: https://adni.loni.usc.edu.

## Author contributions

JT contributed to the conception of the study. JZ and JT performed the experiment. XX and TL contributed significantly to analysis and manuscript preparation. ZC and RC performed the data analyses and wrote the manuscript. JC and XL helped to perform the analysis with constructive discussions. All authors have read and approved the manuscript.

## Conflict of interest

The authors declare that the research was conducted in the absence of any commercial or financial relationships that could be construed as a potential conflict of interest.

## Publisher’s note

All claims expressed in this article are solely those of the authors and do not necessarily represent those of their affiliated organizations, or those of the publisher, the editors and the reviewers. Any product that may be evaluated in this article, or claim that may be made by its manufacturer, is not guaranteed or endorsed by the publisher.
